# Axons Amplify Somatic Incomplete Spikes into Uniform Amplitudes in Mouse Cortical Pyramidal Neurons

**DOI:** 10.1371/journal.pone.0011868

**Published:** 2010-07-29

**Authors:** Na Chen, Jiandong Yu, Hao Qian, Rongjing Ge, Jin-Hui Wang

**Affiliations:** State Key Lab for Brain and Cognitive Sciences, Institute of Biophysics, Chinese Academy of Sciences, Beijing, China; INSERM U901, France

## Abstract

**Background:**

Action potentials are the essential unit of neuronal encoding. Somatic sequential spikes in the central nervous system appear various in amplitudes. To be effective neuronal codes, these spikes should be propagated to axonal terminals where they activate the synapses and drive postsynaptic neurons. It remains unclear whether these effective neuronal codes are based on spike timing orders and/or amplitudes.

**Methodology/Principal Findings:**

We investigated this fundamental issue by simultaneously recording the axon versus soma of identical neurons and presynaptic vs. postsynaptic neurons in the cortical slices. The axons enable somatic spikes in low amplitude be enlarged, which activate synaptic transmission in consistent patterns. This facilitation in the propagation of sequential spikes through the axons is mechanistically founded by the short refractory periods, large currents and high opening probability of axonal voltage-gated sodium channels.

**Conclusion/Significance:**

An amplification of somatic incomplete spikes into axonal complete ones makes sequential spikes to activate consistent synaptic transmission. Therefore, neuronal encoding is likely based on spike timing order, instead of graded analogues.

## Introduction

**Figure 1:**
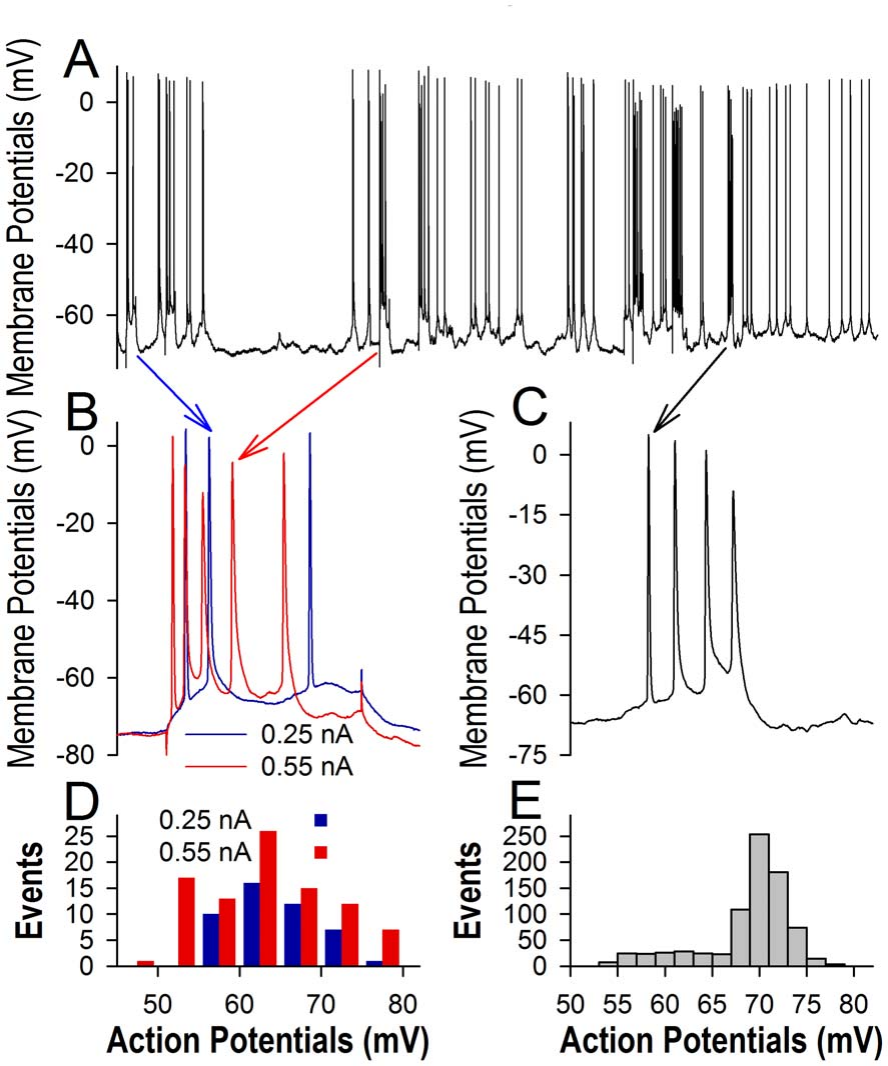
Sequential action potentials vary in their amplitudes. Intracellular recording of spikes was done from mice *in vivo* (**A**). **B**) Sequential spikes evoked by current pulses decrease in amplitudes when the intensity of input currents are raised from 0.25 nA (blue traces) to 0.55 nA (red traces). **C**) Spontaneous sequential spikes decrease progressively in amplitudes. **D**) shows the comparison of sequential spikes evoked by current pulses 0.25 nA (blue bars) and 0.55 nA (red bars). **E**) shows the distribution in the amplitudes of spontaneous spikes in (A).

Sequential action potentials recorded at the soma of most nerve cells in the brain appear variable amplitudes, especially when more spikes are recruited by the enhanced inputs *in vivo* (Fig. 1) or when they are initiated during relative refractory period [1] and membrane potential fluctuation [2]. It is not clear whether these spikes in low amplitudes are securely propagated via the axons and reliably transmitted at the synapses to drive postsynaptic cells, and whether the neuronal output spikes encode different messages based on their amplitudes, i.e., the graded analogues [2], [3], and/or on timing orders, i.e., a “1” (spike) and “0” (spike interval) pattern similar to “on-off” silicon in computer process unit [4], [5]. These questions would be addressed by examining whether somatic sequential spikes are justified to constant levels during axonal propagation, and whether the spikes in different amplitudes induce the consistent patterns of synaptic transmission.

Little is known about the regulations for the axons to propagate sequential spikes except local circuit current is presumed to depolarize cell membrane to threshold for spreading action potentials [6]. Theoretically, to propagate sequential spikes, the axons should be out of spike refractory period before subsequent somatic spikes arrive, i.e., always ready to responding to the spikes coming from their upstream. If it is a case, we predict that the axons possess shorter VGSC-mediated refractory periods, compared with the soma. The shorter refractory periods of axonal VGSCs allow their high density [7] to amplify low amplitude spikes.

To these questions, we conducted pair-recordings between pre- and postsynaptic neurons to test how sequential spikes are reliably transmitted at unitary synapses, as well as between the soma and axon in identical neurons to test whether sequential spikes are propagated on the axon in facilitated manner. We also studied mechanisms underlying spike propagation at the level of single VGSCs.

## Results

### The soma may generate sequential action potentials with low amplitudes

Sequential action potentials vary in their amplitudes. In intracellular recordings *in vivo* (Fig. 1A), the amplitudes of evoked spikes are lowered by raising input intensity (1B) and the amplitudes of spontaneous spikes in bursts appear a progressive decrease (1C). Quantitative data in Figs. 1D–E illustrate that the amplitudes of both evoked (1D) and spontaneous spikes (1E) fall into a wide range, indicating that the neurons may generate action potentials with lower amplitudes, i.e., incomplete spikes, under physiological conditions. This result supports the observation that the spikelets are recorded in the freely moving rats [8].

**Figure 2:**
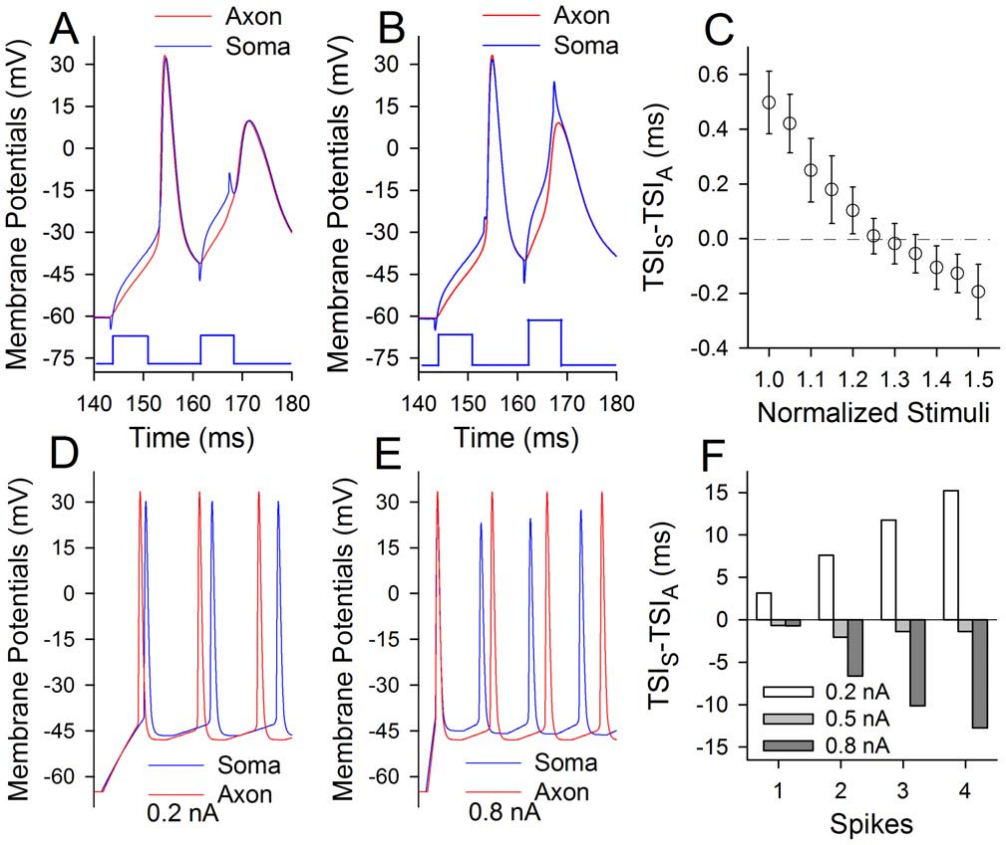
Sequential spikes are initiated at the soma when input intensity increases. In A–C, whole-cell recoding was done at the soma and axon of identical neurons when the paired-current pulses were injected into the soma (blue traces in A–B). **A–B**) show the comparisons in the time of spike initiation (TSI) at the soma (blue traces) and axon (reds), when the intensities of second pulse were low (A) and high (B). TSI for the soma appears moving in front of the axon when stimulus intensity is raised. **C**) shows the difference of TSI at the soma and axon versus the normalized stimulus intensity, in which the negative values of TSI_S_-TSI_A_ indicate somatic spikes ahead of axonal ones. **D–E**) computation-simulated sequential spikes at the soma (blue traces) and axon (reds) when stimulus intensities are 0.2 and 0.8 nA, respectively, in which the strong stimuli switch somatic spikes with decreased amplitudes being ahead of axonal ones. **F**) illustrates the quantitative data of TSI_S_-TSI_A_ for simulated spikes 1–4 under stimulus intensities at 0.2 (white bars), 0.5 (light grays) and 0.8 nA (dark grays), in which the negative values of TSI_S_-TSI_A_ indicate somatic spikes ahead of axonal ones.

We studied whether incomplete spikes were generated at the soma or axon. Simultaneous whole-cell recordings were conducted at the soma and axonal bleb of identical pyramidal neurons in cortical slices. Action potentials at the axon (red traces in Fig. 2) and the soma (blues) were evoked by injecting pair-depolarization pulses (6 ms) into the soma. When the spikes were induced by threshold currents [1], the time of spike initiation (TSI, which is defined as a point of the suddenly rise in *d*V/*d*t, i.e., threshold) at the axon was ahead of that at the soma (Fig. 2A). However, when spikes two were evoked by strong pulses, somatic TSI for spike two was ahead of axonal TSI (Fig. 2B). Fig. 2C shows TSI difference (ΔTSI) between the soma and axon for spikes two vs. the normalized stimuli, in which ΔTSI values shift toward negative when stimulus intensities are elevated (n = 8). This result implies that sequential action potentials are initiated at the soma when input intensity increases.

We then conducted the computational simulation to examine this implication. Fig. 2D–E shows the simulated sequential spikes induced by current pulses at the levels of 0.2 nA and 0.8 nA, respectively, in which strong stimulation induces more spikes with low amplitudes as well as makes somatic spikes being ahead of axonal ones. Fig. 2F shows quantitative analysis for TSI difference between somatic spikes and axonal ones under the conditions of variable stimuli, in which ΔTSI values shift toward negative when stimulus intensity is raised. These results further indicate that sequential spikes with incomplete amplitudes are initiated at the soma of cortical neurons when input intensity increases.

### Somatic incomplete spikes are securely propagated on the axon to activate the synapses

If incomplete spikes are effective neuronal codes, they should activate synapses. If the spikes with various amplitudes encode the different messages, synapse dynamics should be changeable. We examined these possibilities at synapse-coupled neurons, in which sequential spikes were induced at the soma of pyramidal neurons and unitary excitatory postsynaptic currents (uEPSC) were recorded at GABAergic neurons in cortical slices. Incomplete spikes in presynaptic neurons were evoked during relative refractory period (RRP) after the first spikes [1].

**Figure 3:**
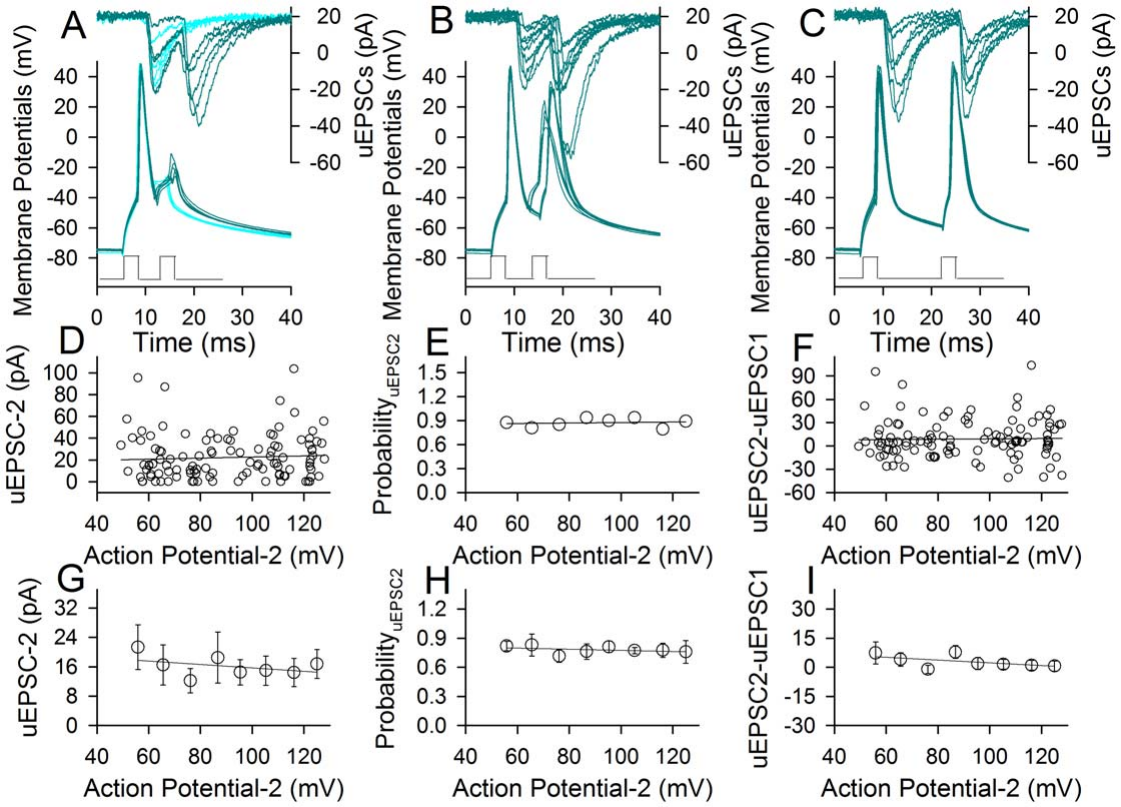
The amplitudes of presynaptic spikes do not influence uEPSC amplitude, probability and patterns. The pair-recording was conducted on presynaptic pyramidal neurons and postsynaptic GABAergic cells. **A–C**) uEPSCs (top traces) are induced by two spikes in a presynaptic neuron (bottom traces). The first spike is normal, and the subsequent spikes are the smallest spikes at ARP (A), incomplete spikes during RRP (B) and complete spikes after RRP (C). Dark green traces show uEPSC two induced by spike two; light blue traces in (A) show only uEPSC-1 induced by spike one. **D–F**) Three plots show relationships between the second spike amplitudes in presynaptic neurons versus uEPSC-2 amplitudes (D), probability (E) and uEPSC2-uEPSC1 (F), respectively, from the experiment in A–C. **G–I**) The averaged data (n = 7) show that the second spike amplitudes in presynaptic neurons do not affect uEPSC-2 amplitude (G), probability (H) and patterns (I). Pair-pulses below panels A–C show the injected currents inducing two presynaptic spikes.

Fig. 3A–C illustrates that incomplete spikes after absolute refractory period (ARP) are sufficient to initiate synaptic activity. The second uEPSCs (uEPSC-2) were induced by spike two after ARP of the first action potentials (dark cyan traces in 3A), during RRP (3B) and after RRP (3C), but not during ARP (cyan traces in 3A). The amplitudes of spike two versus uEPSC-2 are plotted in Fig. 3D–F. There are no the positive correlations between the second spikes and uEPSC-2 amplitudes (3D), probability (3E) or patterns (3F). Data averaged in Fig. 3G-I do not show the effect of spike two amplitudes on uEPSC-2 amplitudes, probability and patterns (n = 7). The results indicate that sequential spikes including those incomplete ones are securely propagated on the axons to be effective neuronal codes, and do not encode different messages to postsynaptic neurons.

The interpretations for this indication include that incomplete spikes to axonal terminals activate the synapses in constant manner or they are amplified in the axon. As the threshold potential for N-/P-type voltage-gated Ca^2+^ channels, which are dominantly localized at axonal terminals, is around −20 mV [9]–[11], the depolarization by the smallest spikes (Fig. 3A) may not be strong enough to activate these Ca^2+^ channels. We examined whether incomplete spikes are amplified on the axons.

### Somatic incomplete spikes are amplified on the axons

The axonal amplification to somatic spikes would be a case, if the inhibition of spikes' propagation in presynaptic axons attenuates the efficacy of synaptic transmission or if the incomplete somatic spikes become bigger in the axons.

**Figure 4 pone-0011868-g004:**
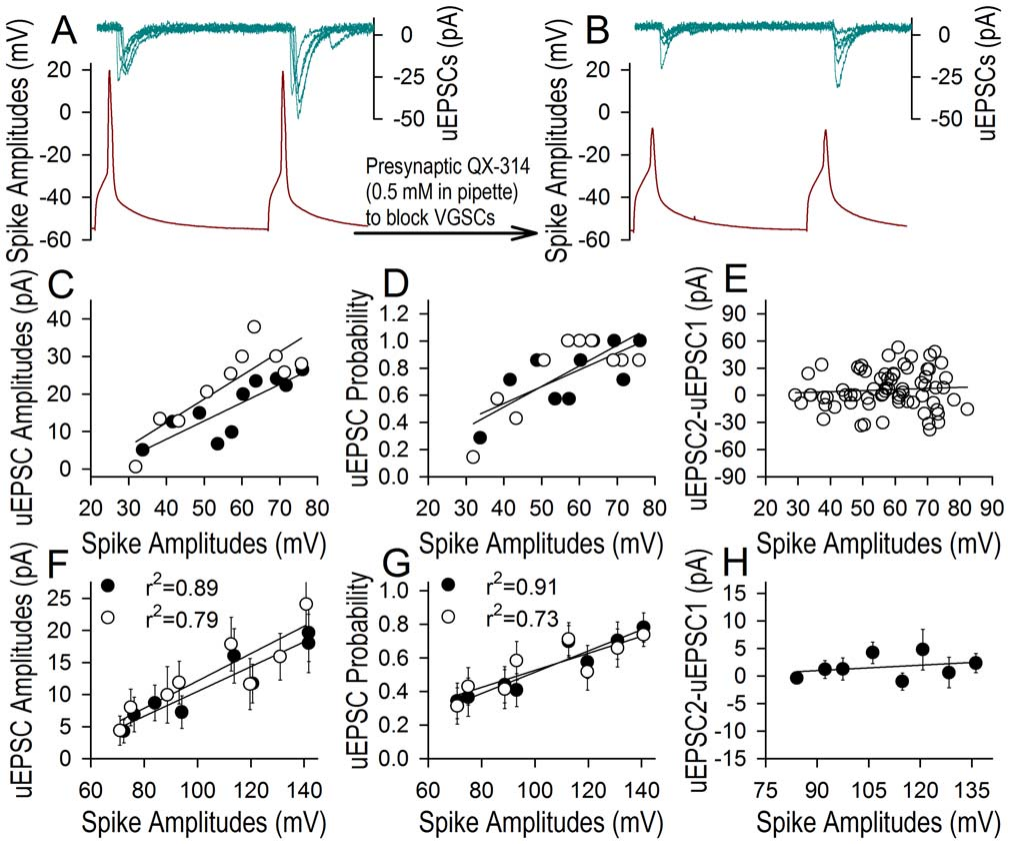
QX-314 lowers spike amplitudes in presynaptic axons and uEPSCs. QX-314 (0.5 mM) was infused into presynaptic neurons through the recording pipettes, when pair-recordings were conducted on presynaptic pyramidal neurons and postsynaptic GABAergic cells. **A–B**) uEPSCs (top traces) are induced by two spikes in presynaptic cell (bottom traces) before (A) and after (B) QX-314 infusions. The amplitudes of both spikes and uEPSCs are reduced after infusing QX-314. **C–E**) Statistical analyses from this experiment show relationships between spike amplitudes and uEPSC amplitudes (C), probability (D) or uEPSC2-uEPSC1 (E). Presynaptic QX-314 infusion decreases spike amplitudes and uEPSCs in a parallel manner. **F–H**) The presynaptic infusions of QX-314 lower spike amplitudes as well as uEPSC amplitude (F) and probability (G) in a linear correlation manner (r^2^ values are in the range of 0.73–0.91, p<0.01, n = 13), except for the patterns (H). Black symbols denote spike one versus uEPSC one, and open symbols are spike two versus uEPSC two.

Spikes' propagation in the axons was inhibited by infusing QX-314 (a VGSC blocker [12], [13] into presynaptic cells through the recording pipettes (0.5 mM). Fig. 4 shows the relationship between presynaptic spikes and uEPSCs, in which uEPSCs (top traces) and spikes (bottom traces) are pair-recorded before (4A) and after (4B) blocking presynaptic spikes. Dynamic changes in spike amplitudes versus uEPSCs from this sample are plotted in Fig. 4C–D, where spikes and uEPSCs in one (filled symbols) and two (open ones) decrease in a parallel way except for uEPSC pattern (4E). Data averaged in Fig. 4F–H illustrate the QX314-induced attenuation of spikes versus uEPSC amplitudes and probability in linear correlations (r^2^ are in a range of 0.73∼0.91). The reduction of synaptic efficacy by blocking presynaptic VGSCs indicates that the axons can amplify spike-induced synaptic events.

**Figure 5:**
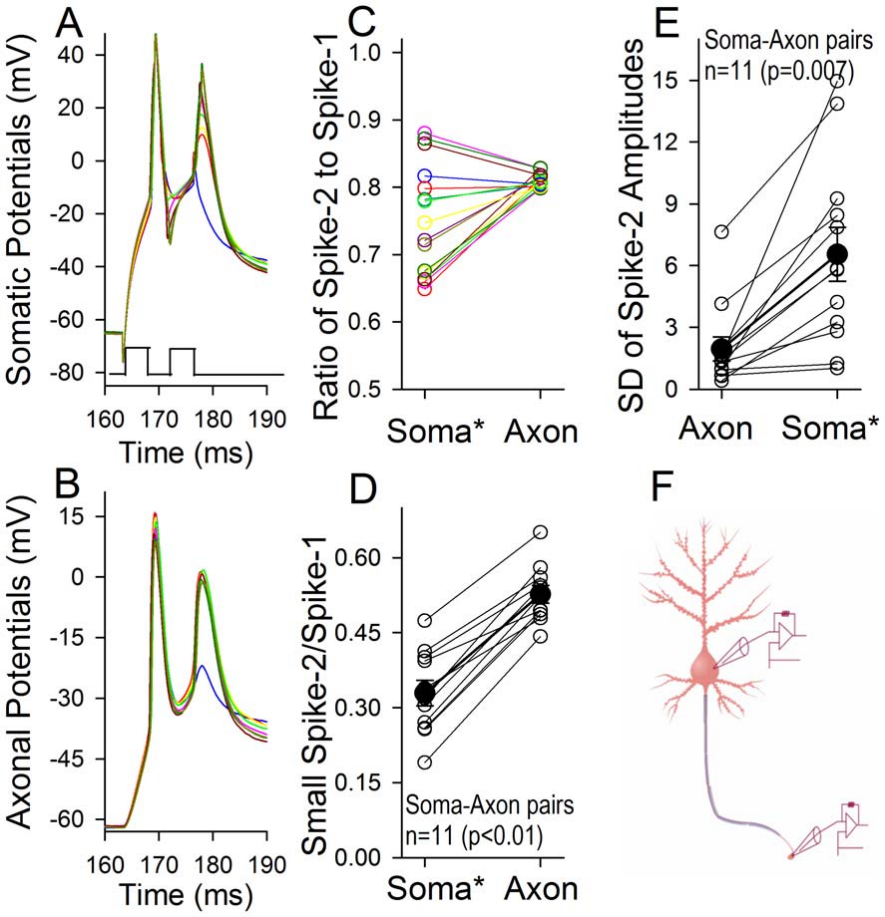
The axons convert somatic sequential spikes to large and constant levels. **A–B**) Whole-cell recordings were conducted at the soma and axonal bleb on the same neurons simultaneously, in which somatic and axonal spikes were induced by somatic current pulses (3 ms). The delay and intensity of the second pulse were adjusted to induce the second somatic spikes in different amplitudes just after ARP. Except for no spike two (blue trace), somatic spike amplitudes vary referred to axonal spikes. **C**) illustrates the ratios of the second spikes to the first ones in their amplitudes (Action Potential-2/-1) that are soma-evoked (*) and axon-corresponded for the sample in A–B. **D**) shows the ratios of the second spikes in the smallest amplitude of soma-evoked spikes (*) to the first spikes versus in the axon-corresponded ones (Spike-2 in the smallest amplitude is divided by Spike-1), 0.33±0.05 for soma-evoked spikes and 0.54±0.02 for axonal ones (n = 11, p<0.001), indicating that the axon amplifies somatic spikes. **E**) shows the standard deviation of the second spikes in amplitude that are soma-evoked (*) and axon-corresponded, i.e., 6.55±1.31 for soma-evoked spikes and 1.95±0.59 for axonal ones (n = 11, p = 0.007), indicating that the axon makes somatic spikes uniform. **F**) a diagram shows simultaneous recoding in axonal bleb and soma of a single neuron. Asterisk (*) denotes the loci of evoking action potentials.

We further examined whether the axons convert incomplete somatic spikes into complete spikes. Action potentials were recorded simultaneously at the soma and axon of identical neurons [14]. Similar to Fig. 3, incomplete spikes were evoked during RRP. As showed in Fig. 5A–C for an example, spikes in various amplitudes evoked at the soma (5A) become larger and constant ones recorded at the axon (5B). The quantitative analysis of spike amplitudes at the soma vs. the axon is given in Fig. 5C, in which the amplitudes of sequential spikes are normalized based on the first spike, i.e., spike two is divided by spike one. We also quantify a conversion of the smallest somatic spikes into axonal ones. The normalized spikes at the soma (0.33±0.05) and the axon (0.54±0.02) are statistically different (n = 11, p<0.001, Fig. 5D), i.e., the axons amplify sequential spikes. Moreover, the standard deviations in the amplitude of spike two are 6.55±1.31 for soma-evoked spikes and 1.95±0.59 for axon- corresponded spikes (Fig. 5E; n = 11, p = 0.007), indicating that the axon makes somatic spikes uniform. It is noteworthy that this result can be repeated under the condition of experimental temperature at 37°C (Fig S1). Together the data in Figs. 3–5, we conclude that the axons amplify sequential action potentials coming from the soma, which enable them in various amplitudes be the digitalized neural codes.

### Axonal VGSCs show short refractory period and high opening probability/currents

**Figure 6:**
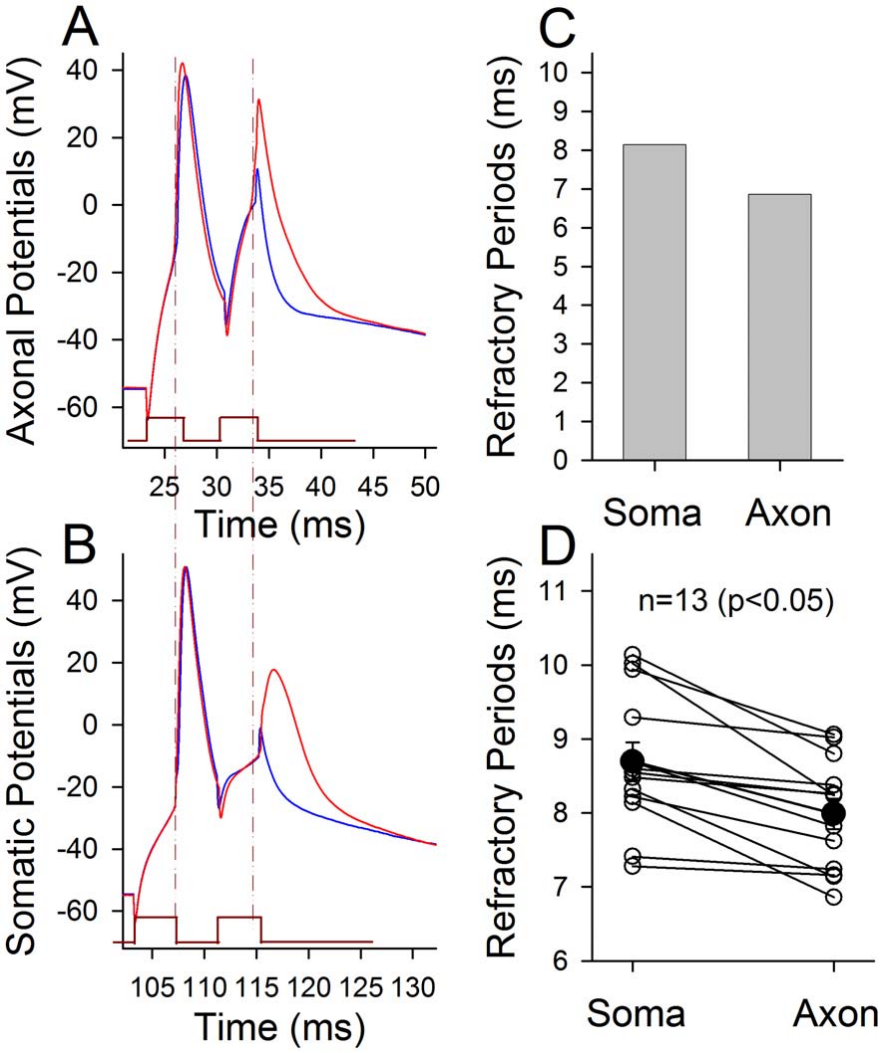
The refractory periods of sequential spikes are shorter at the axons than the soma. Whole-cell recording was conducted at the soma and axonal bleb on the same neurons simultaneously. **A–B**) A sample of soma-axon pairs shows the measurement of the refractory periods of action potentials evoked at axon and soma, respectively, in which current pulses are 3 ms. Dash lines indicate shorter ARP at the axon (A) than the soma (B). **C**) shows axonal ARP is 6.86 ms and somatic ARP is 8.14 ms in this example. **D**) Quantitative data show shorter axonal ARP (7.99±0.21 ms) than somatic ARP (8.7±0.26 ms, filled symbols, n = 13, p<0.05) averaged from individual pairs (open symbols). Asterisk (*) denotes the loci of evoking action potentials.

In terms of mechanisms for the axons to amplify sequential somatic spikes, we propose that the axons are out of spike refractory periods before subsequent somatic spikes arrive, i.e., refractory periods are shorter at the axon than soma. The ARPs of the axon and soma were simultaneously recorded on identical neurons. Fig. 6 shows a comparison of ARPs at the axon versus soma. When the spikes were induced at the axon and soma, respectively, ARPs are shorter at the axon (6A) than soma (6B) as showed by dash lines from an example, in which axonal ARP is 6.86 ms and somatic ARP is 8.14 ms. In averaged data (Fig. 6D), axonal ARP is 7.99±0.21 ms and somatic one is 8.7±0.26 ms (n = 13, p<0.05). Shorter refractory periods enable the axons be always ready to responding to subsequent spikes from the soma. It is noteworthy that threshold stimuli for evoking ARP spikes in Fig. 6A–B are lower at the axon (2.99±0.41 nA) than the soma (6.25±0.75 nA) significantly (n = 13, p<0.01), i.e., the axons are sensitive to subsequent incomplete spikes from the soma. Shorter ARP and lower threshold warrant the axons to amplify somatic spikes.

**Figure 7:**
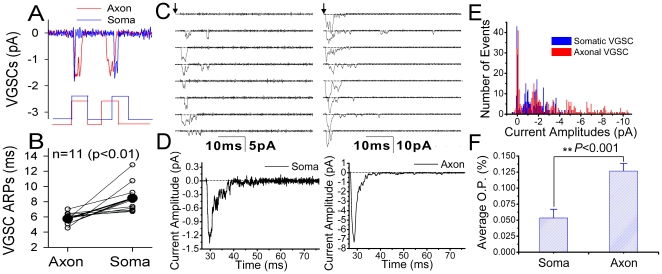
The comparison of refractory period, current amplitude and open probability of voltage-gated sodium channels (VGSC) at the axon vs. soma. Pair-recordings in cell-attached model were conducted at axonal bleb and soma on the same neurons. Two depolarization pulses (5 ms) at thresholds for VGSC activation are given, and the delay of pulse two is adjusted to measure absolute refractory period (ARP) for VGSC reactivation. **A**) Single VGSCs reopen 4.5 ms after ARP at the axon (red line), and 7 ms after ARP at the soma (blue). **B**) shows that the averaged ARP for axonal VGSCs and somatic ones are 5.45±0.2 ms and 8.45±0.54 ms, respectively (n = 11, p<0.01). VGSCs also were activated by 60 ms depolarization pulse. **C**) shows the currents of single VGSCs recorded on the soma (left) versus axonal bleb (right). **D**) illustrates the averaged amplitudes of VGSC currents on the soma (left, 1.35 pA) and axonal bleb (right, 7.3 pA) **E**) shows the distributions in the amplitudes of VGSC currents recorded on the soma (blue bars) and axonal bleb (reds). **F**) shows the opening probability of VGSCs recorded on the soma (left, 0.055±0.015) and axonal bleb (right, 0.127±0.013; p<0.001).

We further tested whether the short refractory period of action potentials at the axon vs. soma is due to the different dynamics of VGSCs since they are inactivated after the spikes [15] in voltage- and state-dependence [16], [17]. Single VGSCs were recorded by cell-attached configuration on the axons and soma of the same neurons, and ARPs for VGSCs' reactivation were measured. Waveforms in Fig. 7A present shorter refractory period to reactivate single VGSCs on the axon (red line) compared to that on the soma (blue). Fig. 7B shows the averaged data of VGSCs' refractory periods at the axon (5.75±0.2 ms) and soma (8.45±0.54 ms; n = 11, p<0.01), which grants shorter ARPs of sequential spikes at the axons showed in Fig. 6.

We also examined whether VGSC open probability and current amplitudes are higher on the axon than soma, which is responsible for the axons to amplify sequential somatic spikes. Single VGSCs were activated by a depolarization pulse (60 ms) and recorded on the soma and axonal bleb of the same neurons in cell-attached model simultaneously. Fig. 7C shows single VGSCs currents recorded on the soma (left) vs. axon (right). The averaged values of VGSC currents from the trails in Fig. 7C are 1.35 pA on the soma (left in Fig. 7D) and 7.3 pA on the axon (right), respectively. In addition, single VGSC currents are lower on the soma (blue bars in Fig. 7E) than axon (red). Fig. 7F shows opening probabilities of VGSCs recorded from the soma (left, 0.055±0.015) and axon (right, 0.13±0.013; p<0.001). Higher opening probability and currents in axonal VGSCs may also be responsible for the axon to amplify sequential somatic spikes.

## Discussion

**Figure 8:**
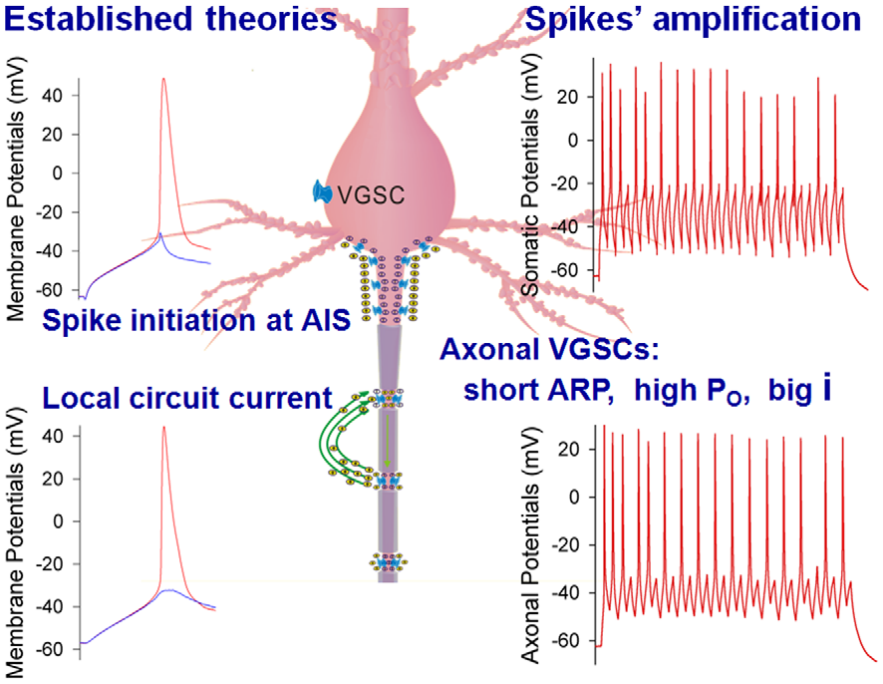
The axons function as spike initiation at AIG and propagation by local circuit currents (established theories in left panels) as well as spikes' amplification (our new finding in right panels). The properties of voltage-gated sodium channels (VGSC) at the axons include shorter absolute refractory period (ARP), higher opening probability (Po) and bigger currents (i), compared with somatic ones. These properties plus local circuit current constitute a comprehensive view for the amplification and propagation of sequential spikes securely at the axons. AIG is an abbreviation for axonal initial segment.

By recording sequential spikes and single VGSCs on the axon versus soma of the same cortical pyramidal neurons simultaneously, we discovered that the axons have shorter VGSC refractory periods (Fig. 6) and higher opening probability/conductance (Fig. 7), compared with the soma. These features make the axons to be out of refractory period before subsequent somatic spikes arrive (i.e., always ready to propagating sequential spikes), and to convert incomplete spikes into complete ones (Fig. 5 & 8). The axonal amplification to incomplete spikes is needed for sufficiently activating N-/P-types of voltage-gated calcium channels at presynaptic terminals because their threshold potentials are −20 mV and above [9]–[11]. With this amplification, all of somatic spikes, especially those incomplete ones generated when spike capacity is raised (Fig. 1), become effective neuronal codes (i.e., real “action potentials”). On the other hand, the longer refractory periods of somatic VGSCs weaken the disturbance of back-propagated axonal spikes to somatic integrations of synaptic inputs. Hence, longer somatic and shorter axonal refractory periods set a rule of downward facilitation and upward attenuation for information flow in the neurons.

Sequential action potentials vary in amplitude *in vivo* and *vitro*, when more spikes are induced by the enhanced inputs (Fig. 1) and when they are initiated during RRP [1]. Do the neurons encode different messages based on spike amplitudes? Compared with dysfunctional axons (Fig. 4), the intact axons convert incomplete somatic spikes toward complete ones (Fig. 5), which drive the synapses in constant dynamics (Fig. 3). In this regard, the neurons write their codes most likely based on the timing orders of action potentials (i.e., “1-0” digital pattern) similar to “on-off” silicon chips in the computer. The decoding of brain program that controls cognition and behaviors should be based on spikes' timing orders, such as their sequences, bursts and clusters. It remains to be examined whether the timing orders of action potentials are solely way for neuronal encoding.

Axonal amplification to sequential spikes generated at neuronal soma also addresses the following issues. Synaptic facilitation is believed to be due to presynaptic residual Ca^2+^ and transmitter release machinery [18]–[21]. By amplifying incomplete somatic spikes to activate N-/P-type voltage-gated Ca^2+^ channels and Ca^2+^-mediated events, the axons ensure the onset of synaptic facilitation (Fig. 3). Second, the neuronal encoding should be precise and reliable for the well-organized cognitions. The neural homeostasis in synaptic patterns and among neuronal compartments improves spike encoding [22], [23]. By amplifying incomplete spikes, the axons will deliver these improved neural codes to their terminals securely. Third, synaptic transmission may be fluctuated (Fig. 3; [23] or be failed [24]. Its mechanism is likely located at the synapses, instead of the failed propagation of spikes on the axons, since Ca^2+^ imaging in cultural neurons demonstrates the propagation of single spikes to axonal terminals [25] and our studies show the secure propagation of somatic sequential spikes through the axons (Fig. 5). Forth, the spikelets recorded intracellularly at cell body *in vivo* [8] may be amplified at the axons and propagated toward their terminals, such that the codes programmed at neuronal soma are always effective signals.

One could argue that shorter refractory periods of axonal spikes let the axons generate sequential spikes easily. This argument is based on the view that the spikes are initiated at axonal hillock [7], [26]–[32]. In fact, the action potentials can be generated at dendrites and soma (Fig. 2; [33]–[41]. Thus, in case of sequential spikes generated at the soma, short axonal refractory periods guarantee the output of soma-encoded spikes through the axons securely.

We reveal that the axons possesses shorter spiking refractory period compared with the soma. This feature enables the axons amplify somatic incomplete spikes to be effective neural codes, i.e., neuronal output codes are based their time order. Our finding updates the functions of the axons to be an amplifier of incomplete spikes, in addition to be an impulse generator at their hillock [7], [27], [29]–[31]. Our finding at the axons in the CNS also broadens the view that the output of action potentials is “all or none”, established at peripheral axon [42]. Moreover, our finding (i.e., the axon as a gainer of somatic spikes) together with the theory of local circuit current [6] present a comprehensive view for the axons to amplify somatic sequential spikes to constant level (Fig. 8) and to propagate them securely toward axonal terminals.

## Materials and Methods

Cortical slices (400 µm) were prepared from FVB-Tg(GadGFP)45704Swn/J mice whose GABAergic neurons express green fluorescent protein (GFP). Mice in postnatal day 15–20 were anesthetized by injecting chloral hydrate (300 mg/kg) and decapitated with a guillotine. The slices were cut with a Vibratome in the modified and oxygenized (95% O_2_/5% CO_2_) artificial cerebrospinal fluid (mM: 124 NaCl, 3 KCl, 1.2 NaH_2_PO_4_, 26 NaHCO_3_, 0.5 CaCl_2_, 5 MgSO_4_, 10 dextrose and 5 HEPES; pH 7.35) at 4°C, and then were held in the normal oxygenated ACSF (mM: 124 NaCl, 3 KCl, 1.2 NaH_2_PO_4_, 26 NaHCO_3_, 2.4 CaCl_2_, 1.3 MgSO_4_, 10 dextrose and 5 HEPES; pH 7.35) 25°C for 1–2 hours before experiments. A slice was transferred to a submersion chamber (Warner RC-26G) that was perfused with normal ACSF for the electrophysiological experiments [43]. The entire procedures were approved by IACUC in Beijing China.

Synapse-coupled pyramidal-to-GFP neurons in layer II-IV of barrel cortex were recorded by a MultiClamp-700B under DIC microscope (Nikon FN-600) simultaneously in whole-cell clamp. Action potentials in pyramidal cells were activated by two depolarization pulses with various intervals at 0.1 Hz. Unitary excitatory postsynaptic currents (uEPSCs) were recorded at GFP-labeled GABAergic neurons under voltage-clamp (holding potential was at −70 mV). The presynaptic spike intervals were set at various values to record uEPSCs during refractory periods, and the intensity of pulses (3 ms) was set to evoke a single spike. Electrical signals were inputted into pClamp-9 (Axon Instrument, Inc, Foster CA, USA) with sample rate at 100KHz. 10 µM CNQX was added to slices at the end of experiments to examine GluR-mediated uEPSCs.

Action potentials on the soma and axonal cutting-end (bleb) of the same neurons were recorded by an amplifier MultiClamp-700B simultaneously with sample rate at 100 KHz to examine the difference of their intrinsic properties. The distances between the recorded soma and axonal bleb were in a range of 70∼150 µm. The judgment for the recordings of these two parts from the same neurons is based on their morphological connection under DIC microscope as well as the “synchrony” presence of the evoked electrical signals. The rationale for not using 100 µM Alexa Fluor 488 [14] to guide the connection between the soma and axon is because we found that Alexa Fluor 488 broadens the duration of action potentials and reduces the amplitudes of sequential spikes.

In our experiments, transient capacitance was compensated, and output bandwidth filter was 3 kHz. Instantaneous and state-steady currents evoked by 5 mV pulses were monitored in all experiments, which were applied to calculate series and input resistance. Standard pipette solution included (mM) 150 K-gluconate, 5 NaCl, 0.4 EGTA, 4 Mg-ATP, 0.5 Tris-GTP, 4 Na-phosphocreatine and 10 HEPES (pH 7.4 adjusted by 2M KOH). Fresh pipette solution was filtered with 0.1 µm centrifuge filter before the use. The osmolarity of pipette solution was 295–305 mOsmol, and the pipette resistance was 6–8 MΩ.

The intrinsic property of pyramidal neurons in our studies was refractory periods after each of spikes. The absolute refractory periods (ARP) of sequential spikes were measured by injecting two depolarization pulses (3 ms in duration and 5% above thresholds in intensity) into the neurons after each of spikes under current-clamp, in which inter-pulse intervals were adjusted. We defined ARP as the duration from a complete spike to a subsequent spike with 50% firing probability [1], [4], [22], [23], [44], instead of the traditional concept for ARP when excitable cells do not respond to stimuli given at the strongest level. Later protocol is not seen under the physiological condition of neuronal activities. Moreover, the use of our measurements for the comparison of ARPs between the soma and axons is logically acceptable.

Intracellular recordings *in vivo* of cortical pyramidal neurons with sharp electrodes were done at mice that were anesthetized by injecting chloral hydrate (300 mg/kg). The electrodes were filled with 2M potassium acetate, and their resistance was in a range of 50–70 MΩ. The signals recorded by AxonClamp-2B in current-clamp model were inputted into pClamp9 with 100 KHz sample rate. In addition to recording spontaneous spikes, we injected depolarization current pulses into neurons to evoke sequential spikes. The data will be analyzed if the resting membrane potential is below −65 mV and the most of action potentials show overshot, which are defined as “complete” spikes.

The currents from single VGSCs were recorded by using MultiClamp-700B/pClamp-9 in cell-attached configuration at the axonal bleb and the soma of identical cortical pyramidal neurons. The pipette solution contains (mM) 120 NaCl, 2 MgCl2, 10 HEPES, 30 TEA and 0.1 mibefradil, where TEA and mibefradil were used to block voltage-gated potassium and type-T calcium channels, respectively. Pipette resistance was 10–15 MΩ. Seal resistance was above 10 GΩ. In recording the currents of single VGSCs, negative voltage pulses were added into the recording pipettes to depolarize membrane potentials. Threshold potentials for VGSC activation were measured by adding the negative voltage pulses (5 ms in duration and 2 mV in each of steps), and refractory periods for VGSC reactivation were measured by changing inter-pulse intervals in 4∼10 ms.

Data were analyzed if the recorded neurons had resting membrane potentials negatively more than −65 mV. The criteria for the acceptation of each experiment also included less than 5% changes in resting membrane potential, spike magnitude, input/seal resistance throughout each of experiments. The input resistance was monitored by measuring cell responses to the hyperpolarization pulses at the same values as the depolarization ones that evoked spikes. The “incomplete” spikes are defined as those without the overshot in amplitudes. Data for single channel recording was taken into account when seal resistance was larger than 10GOhm. The values of spike amplitudes, ARP, uEPSCs and VGSC currents are presented as mean±SE. The comparisons between groups are done by t-test.

Computational simulation for the initiation of sequential spikes at the axon versus soma was conducted based on two components of the evoked action potentials, input integration (subthreshold) and spike impulse. In this regard, total membrane current (I_T_) will be the summation of capacitance current [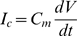] and ionic currents (I_Ion_) The subthreshold component is mechanistically influenced by membrane capacitance (Cm) and leakage (L) currents that affect input resistance. Membrane potentials are determined by an equation, 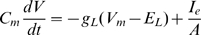 [45]. Spike component is dynamically controlled by the conductance of ionic channels, such as voltage-gated sodium channels (g_Na_m^3^h), voltage-gated potassium channels (g_K_n^4^) and leakage-current channels (g_L_), as described in Hodgkin-Huxley equation, 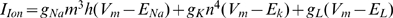 [46]. Based on such factors and the kinetics of single VGSCs obtained from our studies, we wrote the program for the computational simulation of action potentials in Matlab (version 7.0).

In this program, we introduced the values of the parameters, such as time constant (TC) of membrane, threshold potentials (Vts) for VGSC activation, absolute refractory periods (ARP) for VGSC reactivation and hot-spot currents, which were measured from the axon and soma in our experiments. Sequential action potentials simulated by these axonal vs. somatic parameters are given in Fig. 2.

## Supporting Information

Figure S1 The axons convert somatic sequential spikes to large and constant levels. A-B) Whole-cell recordings were conducted at the soma and axonal bleb on the same neurons simultaneously, in which somatic and axonal spikes were induced by somatic current pulses (3 ms). The delay and intensity of the second pulse were adjusted to induce the second somatic spikes in different amplitudes just after ARP. Except for no spike two, somatic spike amplitudes vary referred to axonal spikes. C) Illustrates the ratios of the second spikes to the first ones in their amplitudes (Spike-2/Spike-1) that are soma-evoked and axon-corresponded for the sample in A–B. The experiments were conducted at a temperature of 37°C.
(0.72 MB TIF)
