# Supplementary figures and images for: Axons Amplify Somatic Incomplete Spikes into Uniform Amplitudes in Mouse Cortical Pyramidal Neurons
Source: PLoS One. 2010 Jul 29;5(7):e11868. doi: 10.1371/journal.pone.0011868 (PMC2912328; doi:10.1371/journal.pone.0011868)

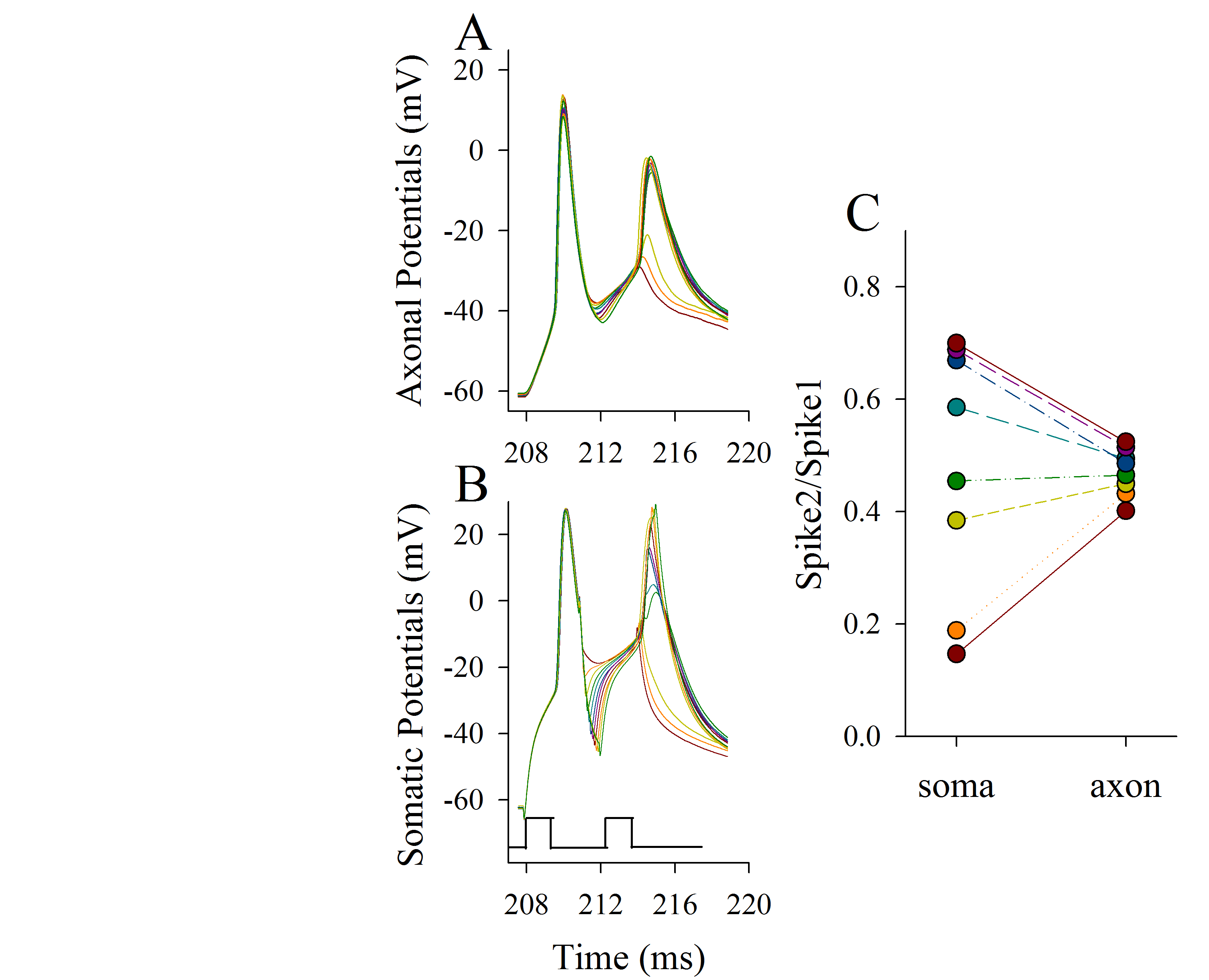

Supplement: Figure S1 — The axons convert somatic sequential spikes to large and constant levels. A-B) Whole-cell recordings were conducted at the soma and axonal bleb on the same neurons simultaneously, in which somatic and axonal spikes were induced by somatic current pulses (3 ms). The delay and intensity of the second pulse were adjusted to induce the second somatic spikes in different amplitudes just after ARP. Except for no spike two, somatic spike amplitudes vary referred to axonal spikes. C) Illustrates the ratios of the second spikes to the first ones in their amplitudes (Spike-2/Spike-1) that are soma-evoked and axon-corresponded for the sample in A–B. The experiments were conducted at a temperature of 37°C. (0.72 MB TIF) [file pone.0011868.s001.tif]
